# Defense System of the Manila Clam *Ruditapes philippinarum* under High-Temperature and Hydrogen Sulfide Conditions

**DOI:** 10.3390/biology12020278

**Published:** 2023-02-09

**Authors:** Yi Liu, Xinmeng Wang, Yanqiu Du, Yi Zhong, Wenguang Wu, Jun Yang, Jihong Zhang

**Affiliations:** 1 National Key Laboratory of Mariculture Biobreeding and Sustainable Production, Yellow Sea Fisheries Research Institute, Chinese Academy of Fishery Sciences, Qingdao 266071, China; 2 Function Laboratory for Marine Fisheries Science and Food Production Processes, Qingdao National Laboratory for Marine Science and Technology, Qingdao 266000, China

**Keywords:** high temperature, hydrogen sulfide, physiological response, *Ruditapes philippinarum*

## Abstract

**Simple Summary:**

During evolution, marine bivalves developed physiological and behavioral strategies to cope with stress. However, the role of behavioral strategies is unclear when the physiological strategies of bivalves contradict behavioral survival and environmental stress. This study presents the effects of high-temperature and hydrogen sulfide conditions on the survival and defensive strategies of the Manila clam *Ruditapes philippinarum*. The results show that both physiological and behavioral strategies play an important role under stress conditions, but the defense system and response strategy of the Manila clam to cope with H_2_S changed with the temperature. This study aims to achieve an understanding of the relationship between the physiological response, behavioral characteristics, and survival of the Manila clam under stressful conditions, and to provide useful information for the culture of the Manila clam.

**Abstract:**

Hydrogen sulfide (H_2_S) acts as an environmental toxin. Despite its toxicity, little is known about the defense strategies of marine bivalves against it. Thus, the tolerance, behavioral characteristics, and physiological response strategies against H_2_S treatment in the sentinel organism Manila clam *Ruditapes philippinarum* were examined. We monitored the survival and behavioral status of Manila clams exposed to different combinations of temperature and H_2_S. The physiological response strategies were examined by measuring the enzymatic activity of cytochrome C oxidase (CCO), fumarate reductase (FRD), superoxide dismutase (SOD), and catalase enzymes (CAT). Moreover, adverse effects of H_2_S on the tissue and cell structure of Manila clams were also examined under a transmission electron microscope. Manila clams responded to H_2_S stress through behavioral and chemical defenses. With exposure to H_2_S alone, Manila clams primarily enhanced aerobic respiratory metabolic pathways in the beginning stages by opening the shell and increasing the CCO activity to obtain more oxygen; with increasing exposure time, when aerobic respiration was inhibited, the shell was closed, and FRD, CAT, and SOD were activated. At this point, Manila clams responded to H_2_S stress through the anaerobic metabolism and antioxidant defense systems. However, high temperatures (≥28 °C) altered the defense strategy of Manila clams. With co-exposure to high temperatures and high H_2_S concentrations (≥20 μmol/L), the Manila clams immediately closed their shells and changed from aerobic respiration to anaerobic metabolism while immediately activating antioxidant defense systems. Nevertheless, this defense strategy was short lived. In addition to this, apparent damage to tissue and cell structures, including mitochondrial ridge dissolution and many vacuoles, was observed in Manila clams exposed to high temperatures and high H_2_S concentrations. Thus, prolonged exposure to high temperature and H_2_S damages the tissue structure of Manila clams, affecting their behavioral capacity and future survival. In summary, profiling Manila clams’ physiological response strategies to H_2_S exposure provided ecological behavioral support for our current understanding of H_2_S detrimental toxicity on marine bivalves.

## 1. Introduction

Due to the rapid development of aquaculture, especially in coastal aquaculture areas, the effects of organic pollutants from human activities on sediment biogeochemical processes have attracted increasing attention [1,2]. The excessive organic load will increase primary productivity and organic matter deposition on the seafloor, thus changing the biogeochemical process of seafloor sediments [3,4]. The oxygen seafloor microorganisms’ requirement to decompose organic matter usually exceeds the surface seawater supply. In summer, when the temperature rises and the water column is thermally stratified or salinity stratified occurs, anoxic conditions develop at the bottom of water bodies and in sediments [5]. Therefore, seafloor hypoxia (dissolved oxygen, DO ≤ 2 mg/L) usually occurs in the summer in eutrophic areas and coastal aquaculture areas worldwide [6]. Low DO concentration in the sedimentary environment promotes anaerobic metabolism and sulfate reduction in sediments, increasing the dissolved sulfide concentration in sediment interstitial water [7].

Diverse toxicities of sulfides at nanomolar to micromolar concentrations in aquatic invertebrates include reversibly inhibiting cytochrome c oxidase (CCO) and enzymes involved in aerobic metabolism [8,9], oxidative stress, and oxidative damage to RNA and DNA [10]. Sulfide includes non-ionized H_2_S, disulfide ions (HS^−^), sulfide ions (S^2−^), and volatile acid sulfide (AVS) [5]. Although sulfides are highly toxic, there is less research on sulfides than on other pollutants [11]. Furthermore, except for non-ionized H_2_S, other sulfides do not show fatal toxicity to aquatic organisms [12,13]. The intermittent surge of H_2_S has a huge toxic effect on oxygen-consuming organisms worldwide, resulting in large-scale death and affecting biodiversity [14]. To better understand the toxic effect of sulfide on aquatic organisms, it is critical to obtain the H_2_S concentration directly. However, research on H_2_S is scarce and superficial at this stage.

Current studies on the adverse effects of sulfide on benthic organisms in coastal areas showed that some benthic organisms that live in environments with high sulfide concentrations have evolved resistance to sulfide toxicity due to the inability to prevent sulfide from entering the body [15]. This ability is thought to result from specialized sulfide detoxification mechanisms, where oxidation of sulfides to less toxic thiol compounds, such as thiosulfates, is achieved by respiratory regulation [9]. Therefore, respiratory and metabolic adaptation may be the main tolerance mechanism for aerobic organisms with tolerance to sulfide. The polychaetes *Hediste diversicolor* and *Marenzelleria viridis* have a strong sulfide tolerance. When sulfide inhibits the activity of their cytochrome c oxidase (CCO), and aerobic respiration cannot be performed normally, their metabolism changes to anaerobic metabolism. This change allows them to use the remaining oxygen in the body for detoxification, while activating fumarate reductase (FRD) to reduce fumarate in the mitochondria to succinate, generating ATP to provide energy [16,17]. In addition to this, aquatic organisms possess antioxidant enzymes to protect their cellular systems from oxidative damage induced by external stimuli [18]. Studies have indicated that when aquatic organisms are exposed to oxidative stress, their antioxidant enzymes’ activities vary with exposure time and pollutant concentration [18,19,20]. Therefore, fluctuations in the activity of antioxidant enzymes, such as superoxide dismutase (SOD) and catalase (CAT), can help us to understand aquatic organisms’ physiological responses.

We speculate that bivalves, which live in benthic environments, also have some ability to cope with H_2_S stress. However, the tolerance and defense strategies of bivalves against sulfide have not been elucidated. Moreover, most studies on the defense strategies of bivalves against environmental stress have focused on physiological response strategies. However, in addition to responding to environmental stress through physiological responses, burrowing bivalves with excavation capabilities can enhance oxidative conditions through bioturbation, which may reduce the possible negative effects of environmental stress in sediments [21,22]. In contrast, at this stage, there are few studies on the effects of environmental stress on bivalve motility behavior. In addition, even when elevated temperatures trigger an increase in sulfide concentration, as compared with the number of studies conducted on the influences of temperature increases or sulfide, the combined effects of increased temperature and high sulfide levels in a controlled environment have been far less studied despite their relevance to marine ecosystems. Moreover, some aquatic organisms known as “sulfide-tolerant”, such as the mudflat polychaete *Glycera dibranchiate*, experience severe cellular damage, impaired tissue proliferation, and altered behavioral abilities when exposed to environmentally relevant sulfide concentrations, even though the animals’ appearance and survival do not appear to be affected [9]. It is currently unknown whether sulfide exposure affects the bivalves’ behavior, particularly behavior such as feeding and digging [11]. Vaquer-Sunyer and Duarte [23] concluded that the fact that aquatic organisms remain alive after exposure to environmental stress does not necessarily mean that they will survive in the long term, as they may have suffered sufficient damage and subsequently die from other causes.

The Manila clam *Ruditapes philippinarum* is an important sentinel organism of seawater pollution and a suitable model species for examining sulfide exposure [5,24]. They are the main benthos living in estuarine beaches and eutrophic coastal areas and the main species of local aquaculture with significant ecological and economic value. Many studies have shown that Manila clams are more likely to be exposed to excessive H_2_S [5,11]. In the present study, we examined the effects of H_2_S exposure on Manila clams to assess their tolerance and behavioral characteristics to different H_2_S concentrations under different temperature conditions, through physiological responses and tissue damage. These results will help us understand the physiological response strategy of the Manila clam under H_2_S stress and the physiological sequelae to provide useful information for Manila clam breeding.

## 2. Materials and Methods

### 2.1. The Experimental Animal

The Manila clams used in the experiment were collected from the Xiaoqinghe Estuary shellfish culture area of Longwei Industrial Co., Ltd., in Weifang City, Shandong Province (37°16.290′ N, 119°04.248′ E). The collection process was performed using a fishing boat suction pump. The collected clams were stored in a clean icebox, kept at 4 °C, and sent to the laboratory within 3 h. The laboratory windows were closed with shading curtains, lighting was turned on only during monitoring, and the light intensity was less than 100 lux to prevent photolysis of sulfide and irritation of Manila clams. All clams were cleaned, numbered, and temporarily kept in a sediment-free tank for 7 days to adapt to the laboratory conditions. The seawater temperature and salinity were controlled at 24 °C (within the optimum temperature range for Manila clams) and 30 ppt, respectively. The clams were fed Chlorella once a day during the temporary feeding period. The water was changed and inflated daily to ensure the temporary aquaculture’s water quality. The clams were divided into 3 groups, and the water temperature in 2 groups increased by 1–2 °C every day according to the experimental design and reached the design temperature before the experiment. The health and activity of the clams were carefully checked before the experiment and only clams with complete, undamaged shells, with a water suction pipe or axe foot protruding from the shell with the ability to retract quickly upon contact with a glass rod, were used for the experiment.

### 2.2. The Experimental Device

**Figure 1:**
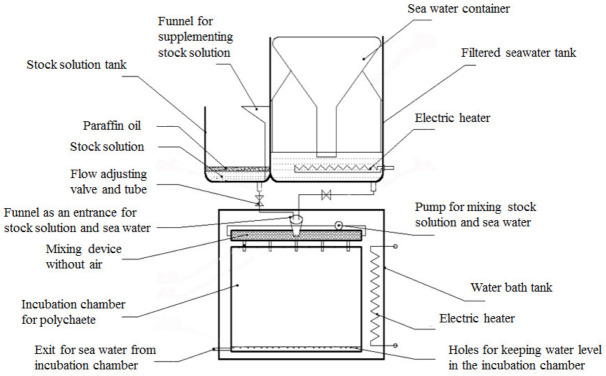
Hydrogen sulfide concentration stabilization system (from Wang et al. [18]).

The experimental setup for this study was modeled after Wang et al. [18], which used a setup that maintained H_2_S stability without overly altering the experimental water quality. The device includes a flow system composed of a sodium sulfide mother liquor tank, a filtered seawater tank, and an experimental tank (Figure 1). The filtered seawater flow rate was adjusted to ensure that the liquid in the experimental tank was fully circulated within 20 min (half-life of H_2_S). The sodium sulfide mother liquor flow rate was adjusted to control different H_2_S concentrations. The filtered seawater and sodium sulfide mother liquor were evenly mixed before entering the experimental tank. Another stock solution tank protected from light was prepared and sealed with paraffin to preserve the stock solution. The stock solution was replenished at regular intervals [18].

### 2.3. The Experimental Design

There were 12 groups of experiments in this study. Each experimental group included 6 parallel groups. A total of 3 groups were used to record death and behavioral characteristics, and the other 3 were used to detect physiological indexes (1–3 Manila clams were randomly selected from each group for analysis at each sampling). A total of 3 groups of temperatures, 24 °C, 28 °C, and 32 °C, were set in the experiment. Under each temperature group, four groups of H_2_S concentrations (0 μmol/L, 10 μmol/L, 20 μmol/L, and 40 μmol/L) were set, and the concentration of 0 μmol/L was used as the control group.

The different H_2_S concentrations were obtained as follows. Firstly, Na_2_S·9H_2_O was mixed with distilled water aerated with N_2_ to prepare the sodium sulfide mother liquor of 160 mmol/L, and then the pH of the mother liquor was adjusted to 8.0 with 1 mol/L hydrochloric acid. Finally, different sulfide concentrations, 0 μmol/L, 80 μmol/L, 160 μmol/L, and 320 μmol/L were obtained by adjusting the flow rates of the sodium sulfide mother liquor and filtered seawater. The real H_2_S concentrations in the experimental tank monitored by the microelectrode system (Unisense, Aarhus, Denmark) were 0 μmol/L, 9.87 μmol/L, 23.85 μmol/L, and 40.82 μmol/L, respectively. The experiment lasted 96 h, and 20 Manila clams (wet weight: 7.35 ± 1.25 g, mean ± SD) were placed in each experimental group.

The mortality and behavioral characteristics were recorded every 6 h after the beginning of the experiment. Samples were taken at 6 h, 12 h, 24 h, 48 h, and 96 h of the experiment period to monitor and analyze physiological indexes. Tissue samples were placed in centrifuge tubes, immediately frozen in liquid nitrogen, and then stored at −80 °C until analysis. After the experiment, the clams were dissected to observe cell structure.

### 2.4. Monitoring Index Analyses

The criteria for judging the mortality of a Manila clam was to touch the suspected dead specimen’s foot or siphon and other body tissues with a glass rod three consecutive times. If there was no response, it was considered dead. The behavior characteristics were obtained by recording the opening and closing behavior of the shell, and the quantitative standard was obtained after modification according to the standard described by El-Shenawy [25] (Table 1). The behavior scores of the surviving clams were recorded, and the total score was divided by the number of surviving shellfish to obtain the opening and closing behavior scores. Each observation was scored 3 times within a 5 min interval. The average value of the three scores was recorded as the Manila clam behavior score. The higher scores indicated that the Manila clam tended to choose to open the shell.

The CCO and FRD in the adductor muscle and CAT and SOD in the hepatopancreas were measured. Tissue samples of adductor muscle were clipped and homogenized by ultrasound in 4 volumes (ratio of buffer volume to tissue weight) of mitochondrial extraction buffer solution (0.25 mol/L Sucrose) at 4 °C. Homogenates were then centrifuged at 600 r/min for 15 min at 4 °C and the supernatants were centrifuged for 30 min (9000 r/min, 4 °C). The precipitate was resuspended with 0.25 mol/L sucrose solution at 4 °C and homogenized again by ultrasound. Homogenates were used for CCO and FRD activity assays. CCO activity was determined by referring to the method of Affonso et al. [26], and FRD activity was determined by referring to Xiao et al. [27]. Tissue samples of hepatopancreas were homogenized individually in 9 volumes of PBS buffer solution (pH 7.2–7.8) at 4 °C. Homogenates were then centrifuged at 11,000 r/min for 10 min at 4 °C. Supernatants for the determination of SOD and CAT were tested within 12 h. The SOD and CAT were analyzed using kits (Nanjing Jiancheng Bioengineering Institute, Nanjing, China). The protein content was determined based on the Coomassie Blue protein assay. Bovine serum albumin (BSA, fraction V) was used for the protein content standard curve.

In this experiment, we randomly selected 3 Manila clams in each experimental group for cellular structure observation. The tissues, including the foot, gill, and adductor muscle of the Manila clam, were sampled for observation by transmission electron microscope as the observation objects. The samples of about 2 × 2 × 3 mm were fixed with 2.5% glutaraldehyde solution and temporarily stored at 4 °C. Then, samples were sliced using a slicer (Leica EM UC7) and embedded. The results were observed under a transmission electron microscope (TEM) (Hitachi HT7700).

### 2.5. Statistical Analyses

The multivariate analysis of variance in R language was used to test the significance of the differences in parameters under different temperatures, H_2_S concentrations, and time. One-way ANOVA was used to examine the effects of H_2_S concentration on the enzyme activities at a given exposure time, and exposure time on the activity score at a given H_2_S concentration. The median lethal time (LT_50_) of Manila clam under different conditions was evaluated according to probit analysis using SPSS 16.0 statistical software (Chicago, IL, USA), and the significance level was set at *p* < 0.05.

## 3. Results

### 3.1. Survival and Behavioral Responses

**Figure 2:**
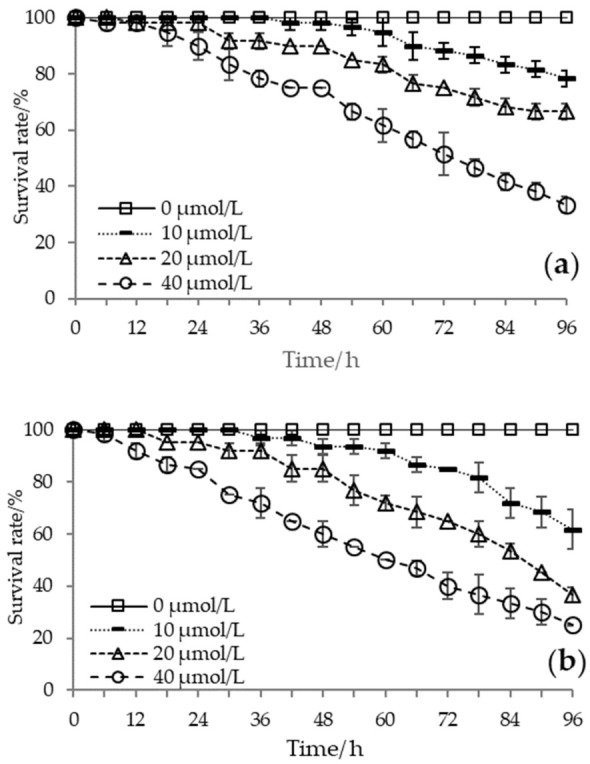

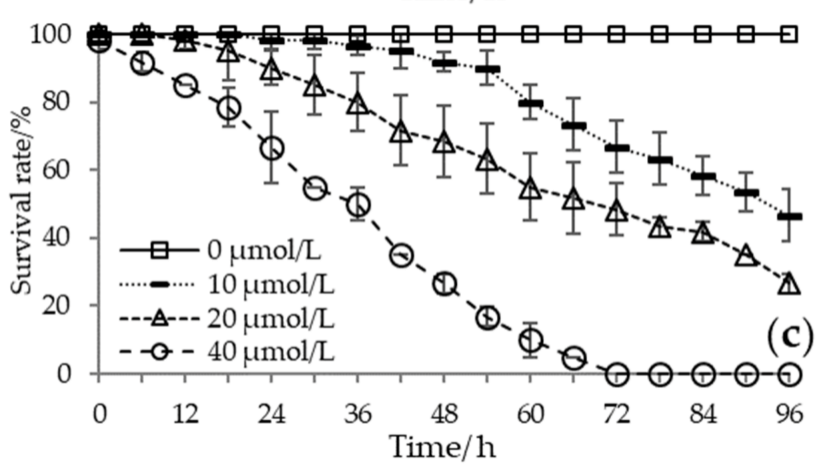
Survival of Manila clams at (**a**) 24 °C, (**b**) 28 °C, and (**c**) 32 °C and under different hydrogen sulfide (H_2_S) concentration conditions (means ± SD, *n* = 3).

The accumulated survival rate decreased with increased temperature and H_2_S concentration, and extinction at 96 h was present only in the most stressed treatments. Meanwhile, individual death began earlier with intensified stress conditions. At 24 °C, batch death was observed after 18 h under the highest H_2_S concentration, whereas in 20 and 10 μmol/L treatments, mortality occurred at 30 and 60 h, respectively. At equal H_2_S concentration conditions, the higher the temperature, the shorter the time to start batch mortality. The behavioral response results showed that mass mortality began when the opening and closing behavior of the Manila clam shifted under high-temperature and high-H_2_S concentration conditions. The survival of the clams was significantly affected by temperature (*F* = 31.849, *p* < 0.01), H_2_S concentration (*F* = 194.923, *p* < 0.01), exposure time (*F* = 360.866, *p* < 0.01), and the combined effect of H_2_S concentration with temperature or exposure time (H_2_S × temperature: *F* = 18.376, *p* < 0.01; H_2_S × exposure time: *F* = 142.030, *p* < 0.01) (Figure 2). According to the probit model analysis, when the H_2_S concentration was 10 μmol/L, 20 μmol/L, and 40 μmol/L, the median lethal times (LT_50_) at 24 °C were 118.45 h, 107.60 h, and 74.29 h, respectively, and the LT_50_ at 28 °C were 104.86 h, 84.36 h, and 63.38 h, respectively, while the LT_50_ at 32 °C were 89.53 h, 70.47 h, and 33.83 h, respectively.

**Figure 3:**
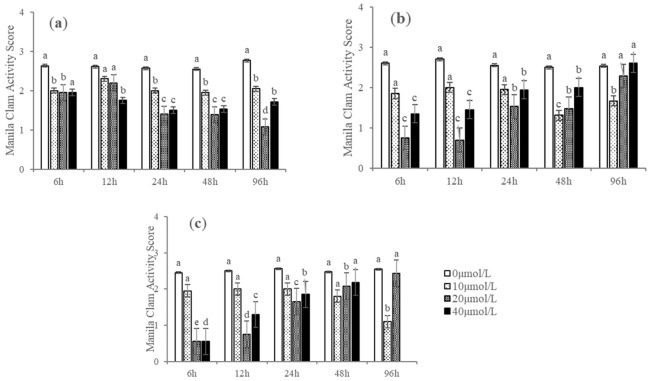
Shell opening and closing behavior of Manila clams at (**a**) 24 °C, (**b**) 28 °C, and (**c**) 32 °C, and different hydrogen sulfide (H_2_S) concentrations (means ± SD, *n* = 3). Bars without shared letters for the same H_2_S concentration indicate significant differences (one-way ANOVA, *p* < 0.05). No data were available at 32 °C and H_2_S = 40 μmol/L at 96 h because all Manila clams died.

On average, H_2_S addition decreased Manila clam activity, but the relationship between activity score and exposure duration differed among concentration and temperature treatments. Completely inactive individuals were observed only in the initial 12 h at higher temperatures (28 °C and 32 °C) and H_2_S concentrations (20 μmol/L and 40 μmol/L). However, this phenomenon lasted for a relatively short period (6–12 h), and the Manila clams’ shells began to open frequently with H_2_S exposure time. At lower temperatures (24 °C) and lower H_2_S concentrations (10 μmol/L), the Manila clams closed their shells tightly more frequently after a certain time of exposure (48 h). The shell opening and closing behavior of the Manila clam was significantly affected by temperature (*F* = 4.110, *p* < 0.05) and H_2_S concentration (*F* = 23.020, *p* < 0.01) instead of the interaction between temperature and H_2_S concentration (*F* = 0.395, *p* = 0.757) (Figure 3).

### 3.2. Physiological Responses

**Figure 4:**
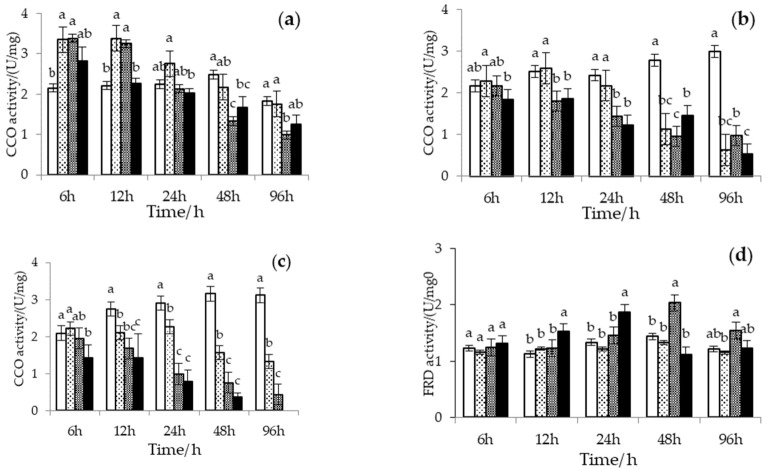

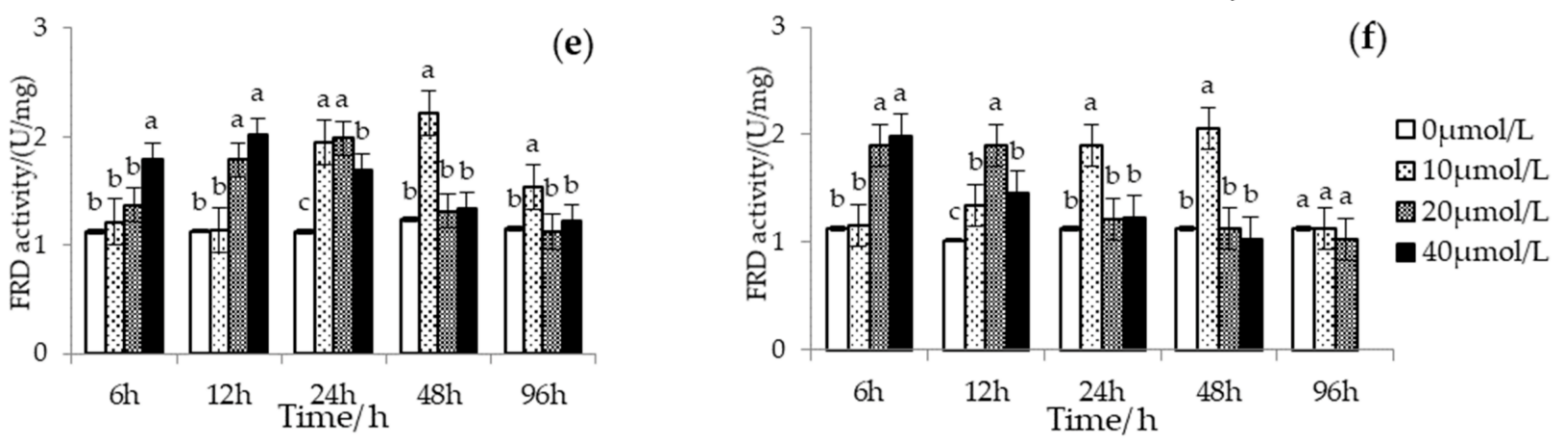
Cytochrome C oxidase (CCO) activity in the muscle tissue of Manila clams at (**a**) 24 °C, (**b**) 28 °C, and (**c**) 32 °C, and Fumarate reductase (FRD) activity in the muscle tissue of Manila clams at (**d**) 24 °C, (**e**) 28 °C, and (**f**) 32 °C under different hydrogen sulfide (H_2_S) stress conditions (mean ± SD, *n* = 3). Bars without shared letters for the same time indicate significant differences (one-way ANOVA, *p* < 0.05). No data were available at 32 °C and H_2_S = 40 μmol/L at 96 h because all Manila clams died.

The CCO activity of Manila clam under high temperature and H_2_S stress is shown in Figure 4a–c. When there was no H_2_S exposure, the CCO activity increased with temperature and exposure time. Adding H_2_S changed this pattern. At low temperatures (24 °C), the CCO activity of the Manila clams increased and then decreased under H_2_S stress. The CCO activity reduction rate was directly proportional to the H_2_S concentration. At high temperatures (28 °C and 32 °C), the CCO activity did not increase significantly with an increase in temperature at H_2_S stress. On the contrary, the downward trend was more evident with increasing temperature and H_2_S concentration. When the temperature was 28 °C, the CCO activity in all H_2_S treatment groups was lower than that in the control group after 48 h of H_2_S exposure. At 32 °C, the CCO activity in all H_2_S treatment groups was lower than that in the control group after 12 h of H_2_S exposure. The multivariate analysis of variance showed that temperature, H_2_S concentration, and exposure time significantly affected the CCO activity of the Manila clam (temperature: *F* = 16.882, *p* < 0.01; H_2_S: *F* = 44.817, *p* < 0.01; exposure time: *F* = 35.998, *p* < 0.01). The interaction between H_2_S concentration and temperature or exposure time also significantly affected the CCO activity (H_2_S × temperature: *F* = 14.147, *p* < 0.01; H_2_S × exposure time: *F* = 7.976, *p* < 0.01).

The FRD activity of Manila clams under high temperature and H_2_S stress is shown in Figure 4d–f. Under the experimental conditions, different temperatures did not significantly affect the FRD activity. With the addition of H_2_S, the FRD activity almost always showed a trend of increasing and decreasing. Furthermore, a decrease in CCO activity accompanied the increase in FRD activity at the beginning. Differently, FRD activity responded immediately and increased significantly at high temperatures (28 °C and 32 °C) and high H_2_S concentrations (20 μmol/L and 40 μmol/L) but was maintained for a short period and started to decrease significantly around 12–24 h. In contrast, FRD activity increased slowly with exposure time and then decreased at high temperature and low H_2_S concentration (10 μmol/L) or low temperature (24 °C) condition. In conclusion, the FRD of Manila clams was significantly affected by H_2_S (*F* = 4.984, *p* < 0.05) and the interaction between H_2_S and temperature (*F* = 5.537, *p* < 0.05) instead of temperature (*F* = 0.038, *p* = 0.845), although it decreased faster at high temperatures.

**Figure 5:**
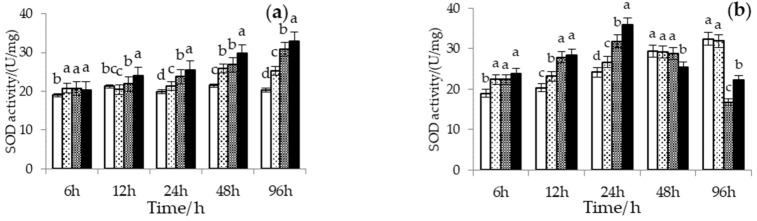

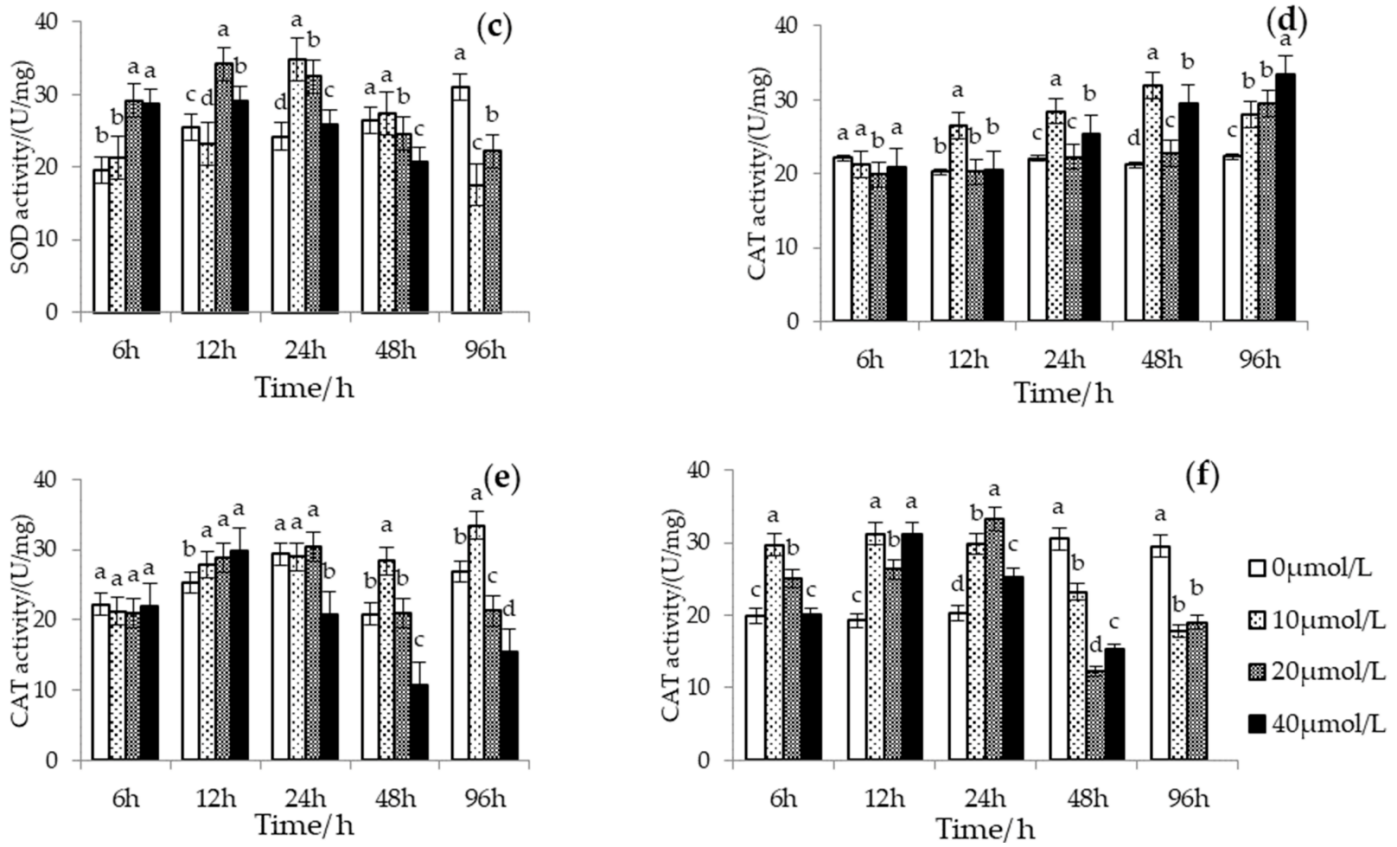
Superoxide dismutase (SOD) activity in the hepatopancreas of Manila clams at (**a**) 24 °C, (**b**) 28 °C, and (**c**) 32 °C, and Catalase activity (CAT) in the hepatopancreas of Manila clams at (**d**) 24 °C, (**e**) 28 °C, and (**f**) 32 °C under different hydrogen sulfide (H_2_S) stress conditions (mean ± SD, *n* = 3). Bars without shared letters for the same time indicate significant differences (one-way ANOVA, *p* < 0.05). No data were available at 32 °C and H_2_S = 40 μmol/L at 96 h because all Manila clams died.

The multivariate analysis of variance showed that H_2_S concentration (*F* = 9.627, *p* < 0.01) and its interaction with temperature or exposure time (H_2_S × temperature: *F* = 15.598, *p* < 0.01; H_2_S × exposure time: *F* = 9.113, *p* < 0.01), instead of temperature (*F* = 2.550, *p* = 0.116) and exposure time (*F* = 0.159, *p* = 0.692), significantly affected SOD activity (Figure 5a–c). At a low temperature (24 °C), H_2_S increased SOD activity in Manila clam with exposure time, and the higher the H_2_S concentration, the higher the SOD activity. Under the combined stress of high temperature (28 and 32 °C) and H_2_S, the SOD activity in Manila clams increased and then decreased. The higher the H_2_S concentration, the earlier the activation of SOD activity and the faster the activity decline.

The multivariate analysis of variance showed that temperature (*F* = 13.696, *p* < 0.01), exposure time (*F* = 9.884, *p* < 0.01), H_2_S concentration (*F* = 43.303, *p* < 0.01), and its interaction with temperature or exposure time (H_2_S × temperature: *F* = 16.919, *p* < 0.01; H_2_S × exposure time: *F* = 7.712, *p* < 0.01) significantly affected CAT activity (Figure 5d–f). The CAT activity of Manila clams responded rapidly at low H_2_S concentrations (10 μmol/L), and increased significantly during the initial exposure time, then began to decrease with increasing exposure time at 32 °C. The activity response of CAT was also rapid at high temperatures (28 and 32 °C) and high H_2_S concentrations (20 and 40 μmol/L), and CAT activity began to decrease with increasing exposure time. The CAT activity reduction rate was directly proportional to the H_2_S concentration.

### 3.3. Cellular Structure Damage

**Figure 6:**
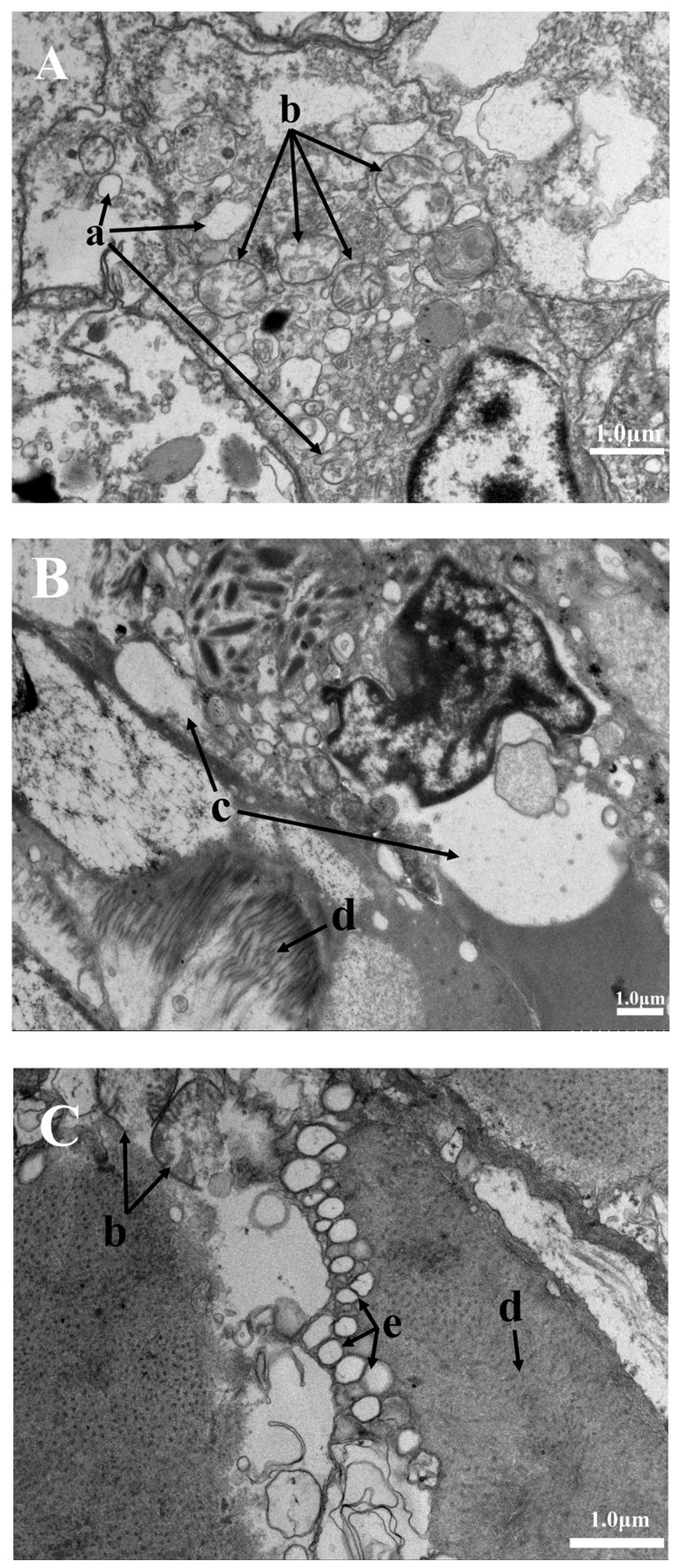
Transmission electron microscope images of the Manila clam muscle. (**A**–**C**) represent the Manila clam’s damaged gills, foot, and adductor muscle, respectively. (a) Indicates a vesicular expanded endoplasmic reticulum. (b) Indicates swollen and vacuolated mitochondria. (c) Indicates large vacuoles in cells. (d) Indicates disordered, blurred, and dissolved myofilaments. (e) Indicates swollen sarcoplasmic reticulum.

The gill, foot, and adductor muscle cellular structures in the 40 μmol/L groups at 24 °C and the H_2_S ≥ 20 μmol/L groups at high-temperature conditions (28 °C and 32 °C) were significantly damaged. Many vacuoles appeared in the cells of each tissue (Figure 6), even in live Manila clams. Mitochondria, the “energy factory” of cells and the principal place of aerobic respiration, appeared to swell and then vacuolized, accompanied by the dissolution disappearance of the ridge. The endoplasmic reticulum vesicles expanded with obvious degranulation. The electron density of the entire cell is low. The muscle filaments of the foot were partly disorderly arranged and dissolved, while that of the adductor muscle was not closely arranged, with a large gap and swollen sarcoplasmic reticulum.

## 4. Discussion

### 4.1. Behavioral Defense to H_2_S Stress

Marine organisms respond to environmental stress in various ways, including behavioral responses [22,28,29]. In this study, Manila clams exhibited different opening and closing behaviors under different stress conditions, suggesting that the behavioral defense strategies of Manila clams change in response to environmental stress. Previous studies proved that bivalves’ shell opening and closing behavior is a rapid and quantifiable index that can be used to evaluate the effects of stress on bivalves [30]. In general, during the initial stage of stress, the opening diameter of the bivalve siphon and the opening and closing degree of the shell are significantly larger than that under normal conditions to obtain more oxygen or food to supplement the energy needed to deal with environmental stress [25]. The bivalve closes the shell for self-protection, and the frequency of the siphon extension decreases with the stress time and state of deterioration. However, our study found that the Manila clams’ shell opening and closing behavior conformed to the above law only at low temperatures (24 °C, H_2_S of 0–40 μmol/L) or at high temperatures but in low H_2_S concentrations (28 °C and 32 °C, H_2_S ≤ 10 μmol/L). This suggests that under low environmental stress conditions, Manila clams prefer to actively obtain more oxygen through respiration or bioturbation in the early stages of exposure to cope with the stress caused by H_2_S and temperature. When the exposure time is continuously prolonged, Manila clams choose passive defense through shell closure. When a relatively high signal was present (28 °C and 32 °C, H_2_S ≥ 20 μmol/L), Manila clams immediately adopted a passive behavioral defense of closing the shell. However, it is important to note that this behavioral defense is not sustainable under high-stress conditions because harsher environmental conditions may lead to the Manila clams’ impaired behavior, meaning that it may no longer be able to close its shell for long periods for self-protection. In this study, when the defensive behavior of the closed shell of Manila began to change, the open shell behavior led to high mortality. These results suggest that the behavioral defense response of Manila clams is rapid under stressful conditions but that differences in stressful conditions alter their behavioral characteristics.

### 4.2. Chemical Defense to H_2_S Stress

Behavior and physiology are inseparable; behavior is the overall response to changes in the external environment and internal physiology [24]. Therefore, different chemical defense strategies of aerobic and anaerobic metabolism under H_2_S stress can explain the altered behavioral defense strategies of Manila clams observed in this study. The H_2_S detoxification process consumes oxygen [31], aggravating the oxygen consumption of the Manila clams. Therefore, in the early stages of exposure, Manila clams improve their respiratory and metabolic intensity by activating CCO activity [17] and maintaining open shell behavior to obtain more oxygen for aerobic metabolism, and the H_2_S in the body is metabolized by the oxidative activity of cellular tissues to less toxic or non-toxic compounds [31]. With the extension of H_2_S exposure time, H_2_S toxicity blocks the aerobic metabolism of bivalves. Excess H_2_S can combine with iron ions, affect the normal operation of the CCO heme porphyrin ring, and inhibit CCO activity, negatively affecting respiratory metabolism [32]. When aerobic metabolism is suppressed, bivalves need anaerobic metabolism to provide energy [33]. At this time, the Manila clam adopts the chemical defense of anaerobic metabolism while performing the behavioral defense strategy of shell closing to prevent H_2_S from entering the body as much as possible. Likewise, the chemical defense strategy of Manila clams changed under high-temperature conditions. Although increased temperature enhances aerobic respiration and metabolic intensity of Manila clams, under the synergistic effect of high-temperature and H_2_S conditions, Manila clams may actively close their shell and reduce the intensity of aerobic respiration and metabolism, turn to anaerobic metabolism, reduce the oxygen consumption in the metabolic process, and use the remaining oxygen for H_2_S detoxification. In a related study, *Urechis unicinctus* was found to employ the same anaerobic metabolic strategy in response to high-sulfide concentration conditions [17]. However, the present study indicates that the anaerobic metabolism process of Manila clams is blocked early under the synergistic effect of high-temperature and H_2_S conditions, causing an energy deficit that prevents them from effectively providing behavioral or chemical defenses and ultimately leading to mortality.

In addition to regulating respiratory metabolism, the antioxidant system also plays an important role in the response of marine mollusks to various oxidative stresses [19,20,34]. Antioxidant enzymes, such as SOD and CAT, can be significantly affected by H_2_S and are used as indicators of immune status under H_2_S stress [18,35]. It is believed that H_2_S can change the organisms’ antioxidant activity by inhibiting functional enzymes [36]. Our study found that the CAT activity is more sensitive to low H_2_S concentration (10 μmol/L), consistent with Wang et al. [18]. Previous studies have shown that benthos needs a longer time to eliminate ROS by activating SOD and CAT under high-H_2_S concentration stress [18]. In the present study, SOD and CAT were not immediately activated at high-H_2_S concentrations (≥20 μmol/L) alone, indicating that H_2_S did not affect Manila clams’ antioxidant system during the early stages of exposure, as behavioral and other chemical defenses alleviated the H_2_S-caused stress. However, when the temperature increased, SOD and CAT were immediately activated by the dual effect of high temperature and high-H_2_S concentrations. The SOD and CAT activities started to decrease with exposure time. This may be because different benthos species have different physiological response strategies to the synergistic effects of high-temperature and H_2_S conditions. Manila clams protect the body from the toxic effects of high-temperature and H_2_S conditions by synergistically regulating the respiratory metabolic detoxification and antioxidant systems. Therefore, our results suggest that SOD and CAT play important roles in the Manila clams’ antioxidant defense in the early stage of high-temperature and high-H_2_S concentration exposure. However, the immune regulatory system of the Manila clam was disrupted, and the antioxidant system was damaged to different degrees with the exposure time, which further destroyed the cell biofilm system, resulting in damage to cell structure and function [11].

### 4.3. Organ Specificity in H_2_S Damages

Our results also indicated that the mitochondria of the Manila clam have noticeable ridge dissolution under the combined action of high-temperature and H_2_S conditions. The ridge is the place for many critical biochemical reactions and provides attachment sites for many essential enzymes in the metabolic process. Ridge dissolution may be one key reason that affects Manila clams’ survival at the cellular level [33]. In addition, there was some vacuolation, which the toxicity of H_2_S might cause. The vacuolation reflected the metabolic disorder of the Manila clam to some extent and suggested that their tolerance to environmental stress began to decline. At this time, although there seems to be no change in the survival and appearance of the Manila clam, again, small fluctuations in environmental factors, such as temperature or dissolved oxygen, might increase mortality.

The tissue damage results prove that environmental stress has an “sequelae” on the Manila clam, which may affect future survival, and demonstrates that except for the lack of energy that causes the failure of the clams to close the shell adequately, adductor muscle tissue damage is also a cause of abnormal shell closing behavior. We speculate that tissue damage impaired behavioral capacity leading to the inability to engage in normal digging and feeding. This might result in losing their ability to obtain oxygen through bioturbation, improve the burrow microenvironment, or reburial. Therefore, potentially increasing the clams’ vulnerability to predators [37].

It should be noted that we did not observe tissue damage on the Manila clam at different time points. In the following work, we should augment the observation frequency to clarify the time node of tissue damage caused by different conditions and to establish an early warning mechanism to prevent the irreversible effects of prolonged environmental stress on bivalves.

### 4.4. Synergistic Effect of High Temperature and H_2_S

Our study found that within the known tolerable temperature range of 24 °C to 32 °C [38], increased temperatures and H_2_S concentrations had more severe and damaging effects on Manila clams. The temperature increase, whether from 24 °C to 28 °C or from 28 °C to 32 °C, seemed to be more stressful than the increase in H_2_S concentration. Manila clam mortality at high temperatures and low-H_2_S concentrations was higher than at low temperatures and high-H_2_S concentrations. The synergistic effect of high temperature and H_2_S leads to a greater threat to the survival of the Manila clam. The self-strengthening effect of high temperature may significantly reduce the ability of the Manila clam to deal with H_2_S stress. Increasing temperature is believed to decrease other environmental stress thresholds for many species [38]. Our results also suggest that high temperature and H_2_S in combination have a detrimental effect on Manila clams in terms of mortality and that these two stressors affect clam physiology in different ways. The temperature rise may cause more significant stress than the H_2_S rise within a specific range because the high temperature reduced the H_2_S range, which the Manila clam could tolerate. This may be because elevated temperatures alter Manila clams’ behavioral characteristics, physiological response strategies, and immune defense systems, affecting their survival.

## 5. Conclusions

Under the environmental stress of high-temperature and H_2_S conditions, the Manila clam responds rapidly to these threats by adopting chemical and behavioral defenses. Some chemical defenses, such as the adjustment of respiratory and metabolic strategies, are reflected in behavioral traits. Metabolic and immune regulation in the chemical defense strategy work together to defend against the toxicity of H_2_S. However, increased temperature changes the defense strategy of the Manila clam in response to H_2_S, including changes in shell opening and closing behavior, respiratory, metabolic regulation, and immune regulatory response strategies. Under prolonged environmental stress, some damage to the tissue structure occurs; this damage explains the Manila clams’ altered behavior and demonstrates the “sequelae” of prolonged environmental stress on the Manila clam. In conclusion, a combination of high-temperature and H_2_S stressors is expected to reduce the likelihood of population survival much more than changes in a single stressor. High temperature is not an independent stressor as it also causes an increase in H_2_S in local environments; thus, these stressors should be considered in combination.

## Figures and Tables

**Table 1:** Criteria used for scoring Manila clam activity.

Criterion	Score
The shell is entirely closed or slightly open, but the mantle is not clear	0
The shell is open, and the mantle is visible	1
The shell is open, and the siphon is protruding, but the protrusion length is short	2
The shell is open, and the siphon extends more than 1/3 of its full length	3
The shell opens, and the siphon and foot are extended	4

## Data Availability

The datasets during and/or analyzed during the current study are available from the corresponding author on reasonable request.
